# Can the establishment of free trade zones promote the internationalization of enterprises? – Evidence from micro-enterprise level

**DOI:** 10.1371/journal.pone.0322125

**Published:** 2025-07-16

**Authors:** Jijun Zhang, Haowei Wang

**Affiliations:** International Business School of Hainan University, Haikou, Hainan, China; Southwestern University of Finance and Economics, CHINA

## Abstract

Based on microdata of A-share listed companies in Shanghai and Shenzhen from 2009 to 2022 as research samples, this paper takes the implementation of Free Trade Zones policy as the starting point and using the difference in differences method to analyze the impact of the establishment of Free Trade Zones on the internationalization level of enterprises. The empirical results indicate that (1) Free Trade Zones policysignificantly improved enterprises’ internationalization level, and the conclusion still holds after a series of endogeneity and robustness tests. (2) Mechanism analysis showedthat digital transformation, resource allocation efficiency, and green innovation are intermediary variables affecting enterprises’ internationalization levels in Free Trade Zones. The establishment of Free Trade Zones can promote digital transformation, resource allocation efficiency, and green innovation of enterprises, thereby enhancing their internationalization level. (3) Heterogeneity analysis found that establishing free trade experiments has a more significant effect on improving the internationalization level of enterprises in low city levels, large-sized companies, capital-intensive and technology-intensive companies, businesses with executives who have overseas experience, and high-tech companies (4) Further research has found that the establishment of Free Trade Zones also has a positive spatial spillover effect on enterprises in surrounding cities. The above conclusion enriched and expanded the research on the impact of the establishment of Free Trade Zones on the internationalization level of enterprises and has important practical significance for enhancing the competitiveness of enterprises.

## 1. Introduction

High-level openness is key to achieving Chinese-style modernization. In the new era context, China is working to establish a high-quality economic structure, and the strategy of Development of internationalization for businesses is crucial. As the core means for companies to Development of internationalization, international business operations can help enterprises reduce costs, expand profit margins, and enhance core competitiveness in an environment of increasing global competition and cooperation, thus driving sustainable business development. As a result, many manufacturing companies are choosing to venture into international markets [1].

China has embraced a new phase of high-level openness as a strategic guideline, promoting comprehensive reforms and enhancing external transparency. This marks the start of an era characterized by significant openness. A key aspect of this high-level openness is the establishment of Free Trade Zones, which bring together various domestic and international resources within the same geographic area through a series of institutional innovations. These innovations gradually reduce or eliminate government restrictions on cross-border economic activities [2], aligning with global high-standard trade and investment rules and facilitating the ease of trade and investment [3]. In this way, China has achieved high-level openness at the institutional level, providing the necessary conditions for domestic companies to engage in international cooperation and integrate into global innovation networks.

The establishment of Free Trade Zones(FTZs) is based on constructing an investment environment free of barriers, creating a favorable business environment, and establishing a sound property rights protection system. These measures stimulate institutional reform capabilities and attract foreign investors to engage in investment and trade [4]. The policies implemented by FTZs provide comprehensive support for companies’ internationalization, not only playing a role in attracting foreign investment but also promoting the globalization of domestic enterprises. These policies help businesses better respond to changes in the global economic environment and promote their internationalization process.

This study makes several significant contributions to the existing literature. First, it addresses a research gap by providing empirical evidence on the impact of Free Trade Zones on the international expansion of Chinese firms. By adopting an “outward expansion” perspective, this study conducts a quantitative analysis of how FTZs policy affect firms at the micro level, which sets the stage for future research to evaluate the broader implications of FTZs. Second, we treat the establishment of FTZs as a quasi-natural experiment and categorize cities into groups—designating pilot cities as the treatment group and non-pilot cities as the control group. This methodology allows us to apply the difference-in-differences (DID) method to identify the causal relationship between FTZs establishment and firm internationalization. In comparison to previous studies, our research offers a more in-depth understanding of the effectiveness of FTZs implementation, thereby strengthening the foundation for assessing its economic consequences. Third, this study enriches the literature by identifying three key mediating mechanisms through which FTZs facilitate international expansion: digital transformation, resource allocation efficiency, and green innovation. We also conduct heterogeneity analyses across different firm groups to explore the varying impacts of FTZs policy Finally, our analysis extends the literature by revealing the spatial spillover effects of FTZs on firm internationalization, providing new insights into the broader economic influence of high-level opening-up policies.

## 2. Theoretical background

### 2.1. Institutional background

Since the reform and opening-up, China has made significant achievements in external openness. Especially after joining the World Trade Organization (WTO), China’s institutional framework for external openness has been greatly enhanced and improved, accelerating at an unprecedented pace. With the advancement of the Belt and Road Initiative and the successful signing of the Regional Comprehensive Economic Partnership (RCEP), China has continuously pursued a higher level of openness.

High-level external openness is a key driving force behind the internationalization of businesses. In this context, establishing a domestic economic system that aligns with international economic and trade rules and optimizing the domestic business environment have become urgent tasks. Trade barriers, inefficient regulation, and poor tax efficiency in non-FTZs severely hinder the effectiveness of market governance mechanisms and stifle market vitality. Against this backdrop, FTZs have emerged as testing grounds for China’s new round of economic system reforms. These zones have made remarkable progress in reforms related to trade facilitation, investment management systems, financial system innovation, and government management models. Key systems and mechanisms, such as the Sole Window of system, the negative list management approach, and the “open at the front, manage at the back” regulatory system, have been efficient and could serve as valuable models for nationwide implementation in the future [5].

### 2.2. Theoretical analysis

The literature related to this study involves the following two categories. The benefit analysis of the establishment of FTZs, and the study of factors affecting the internationalization of enterprises.

#### 2.2.1. Research on the benefits of the construction of FTZs.

In recent years, academic research on the establishment of FTZs has deepened, with existing studies primarily focusing on two areas: First, from a macroeconomic perspective, research examines the impact of FTZs policy on macroeconomic development and overall economic performance; second, from a micro-level perspective, studies analyze the effects of FTZs policy on corporate innovation, corporate governance, and business performance.

At the macroeconomic level, existing research confirms that the establishment of FTZs promotes economic growth at the regional [5] and city levels and positively impacts the development of cross-border e-commerce in cities [6]. Additionally, economists have found that the establishment of FTZscan enhance local cities’ innovation capacity, thereby improving local green total factor productivity [7]. Furthermore, the establishment of FTZs can optimize export structures, improve trade efficiency [8], and drive the upgrading of export industries through optimizing human capital structures [9]. These changes enhance the competitiveness of export products, contributing to export growth and facilitating the achievement of the Development of internationalization goal [10].

At the microenterprise level, the establishment of FTZs has significantly improved enterprises’ sustainable development performance within the zones [11]. Institutional innovations brought about by these reforms are also key factors influencing business behavior [6]. Additionally, the institutional environment changes driven by FTZs policy have improved the external financing environment for businesses, increasing the total amount of external financing [12]. Moreover, the establishment of FTZs can empower businesses’ digital transformation by facilitating information flow and risk management and creating favorable conditions. This has played a pivotal role in driving the digital transformation of enterprises [13]. Furthermore, the establishment of FTZs has also attracted foreign enterprises [14] and facilitated the development of Foreign Direct Investment (FDI) [15].

Existing literature has extensively explored the impact of the FTZ establishment and the factors influencing firm internationalization, providing valuable insights into the critical role of FTZs in further expanding China’s opening-up and deepening economic reforms. However, there is still no consensus in academia regarding the effect of the establishment of FTZs on firm internationalization, and the logical connection between the two remains incomplete.

#### 2.2.2. Research on enterprise internationalization.

Enterprise internationalization refers to the process in which a company’s sales and production activities gradually expand from the domestic market to international markets [16]. This signifies a transformation from a local company to a multinational corporation, entering the wave of globalization and conducting business activities in various countries and regions. Whether a company chooses to “go global” is the result of multiple factors working together. After the academic community has explained the theoretical connotations, characteristics, and economic systems of enterprise internationalization, future research will focus on the paths to internationalization and the driving factors behind it.

Looking at the existing literature, research on the factors influencing enterprise internationalization can be roughly divided into two categories. The first category focuses on internal factors such as top executives [17], board members [18], and ownership structure [19], and their impact on enterprise internationalization. The second category examines external factors such as policy [20], market environment [21], and international conditions [22], and their influence on the internationalization of enterprises.

While some studies have begun to examine the relationship between the establishment of FTZs, firm exports, and outward investment, the core objective of China’s FTZ initiative is not merely to promote exports and foreign investment. Instead, it aims to align with high-standard international trade and economic rules through institutional innovation and reform. Second, most prior research has relied on provincial-level data, treating entire provinces as FTZs without considering that only a few specific cities within each province are designated as FTZs. This provincial-level approach may fail to capture the underlying microeconomic mechanisms and could introduce estimation bias when assessing the policy effects of the establishment of FTZs.

Given these gaps, this study focuses on whether high-level opening-up policies, represented by the establishment of FTZs, can effectively promote firm internationalization. By addressing these research shortcomings, our study aims to bridge the existing gaps in the literature and provide a more comprehensive understanding of FTZs policy’ impact on firm internationalization.

## 3. Research hypothesis

### 3.1. Analysis on the direct impact of the establishment of FTZs on the internationalization of enterprises

In recent years, the increasing volatility of the international market environment and the expansion of uncertainty have raised the risks companies face during their international expansion, impacting their intention to adopt internationalization strategies [23]. In this context, institutions and policies have become key drivers for the international development of Chinese enterprises, with the establishment of FTZs providing a solid institutional foundation for companies’ development of internationalization of strategies. Like other factors of production, institutions are a form of productive force, and companies’ economic performance and business activities vary under different institutional frameworks [24,25]. Institutional openness is at the core of China’s progression toward a higher level of external openness, laying a solid foundation for implementing a high-quality, high-level external openness strategy. The establishment of FTZs represent one of the forms of institutional openness. The establishment of FTZs offers a comparative advantage in internationalization [26], enabling companies to better engage in global operations through sound institutional arrangements.

The establishment of FTZs provides an enabling institutional environment for promoting trade liberalization and investment facilitation. It has brought about effective reforms in key systems and mechanisms, such as trade facilitation, investment management, financial system innovation, and government management models [7]. Various measures, such as commodity trading platforms, negative list management, free trade accounts, and social credit systems, have been explored, promoting the free flow of domestic and foreign production factors, achieving efficient resource allocation, and deepening market integration. This accelerates the development of a modern market system aligned with international rules. This provides companies with more capital for complex international operations. Enterprises in the FYZs are encouraged by various preferential policies. They are both willing and able to explore overseas markets. This actively drives their internationalization process. At the same time, FTZs with high levels of openness promote knowledge exchange and technological innovation [27]. Innovation strategies should focus on utilizing a company’s internal resources. Small and medium-sized enterprises can continually improve and develop their resources through innovation. This enhances their competitiveness and impacts their internationalization performance.

The establishment of FTZs can optimize export structures and enhance trade efficiency [8], promote the upgrading of export industries [9], and improve the competitiveness of export products [16], thereby driving export growth and supporting companies in implementing the development of internationalization of strategy. At the same time, the establishment of FTZs provide a more favorable development environment and a positive political and economic atmosphere for companies within the zone, creating favorable external conditions for corporate growth, ensuring smooth operations, and reducing unnecessary costs. The sound financial environment offers diverse financing channels, improving corporate financing capacity, alleviating financing constraints, and assisting in internationalizing business operations. A strong rule of law environment ensures enterprises’ healthy and stable development and lays the foundation for internationalization. The establishment of FTZs provides Chinese companies with a platform to expand their international influence, enter global markets, and establish strategic partnerships with foreign companies. Engaging in international competition through internationalization strategies has become an inevitable choice for Chinese enterprises.

The establishment of FTZs create conditions for business clustering and provide a platform for this concentration. Institutional innovation and policy incentives encourage businesses to concentrate on specific areas. Due to the agglomeration effect, many companies within the same industry and related upstream and downstream industries coexist in the FTZs. This generates internal and external economies of scale, helping companies reduce production costs and access advanced knowledge and technology, promoting their internationalization. Internationalization is how businesses fully utilize global resources and expand into world markets. In this process, knowledge and technological advantages are incredibly crucial. By embedding themselves in FTZs, companies can leverage the zone’s network to acquire knowledge and technology, creating resource-sharing effects. The concentration of capital within the zone provides funding support for innovation, while the zone itself serves as a platform for resource acquisition. The establishment of FTZs create favorable conditions for frequent communication between businesses. Advanced management practices, expertise, and technology spread across companies, promoting internationalization.

Based on this, this paper proposes the research hypothesis H1: The establishment of FTZs has a positive promoting effect on the internationalization of enterprises.

### 3.2. Analysis of the mechanism of promoting the internationalization of enterprises through the establishment of FTZs

#### 3.2.1. Digital transformation mechanism.

The establishment of FTZsand the digital age are progressing in parallel, driving a new round of transformation for Chinese enterprises. The significant changes triggered by digital transformation have become a key factor in driving business development. Enterprises are the micro components of the macroeconomy, and digital technologies can improve business operations’ efficiency, enhance new projects’ development, and increase competitiveness, thus giving enterprises a competitive edge in the global market [28].

As an important testing ground for reform and opening up, FTZs have created favorable policy support, industrial conditions, and market environments for the digital transformation of enterprises [29]. Through mechanisms such as information transmission, risk management, and the creation of enabling conditions, the establishment of FTZs empower businesses to undergo digital transformation. The establishment of FTZs also facilitates the flow of goods and production factors within the zones, enhances enterprises’ research and development capabilities and production efficiency, alleviates financing constraints, improves industrial agglomeration levels, and promotes the digital transformation of businesses, thereby enhancing their digital capabilities [12]. Several studies have confirmed that applying digital technologies can reduce agency costs, ease financing constraints, and enhance enterprises’ ability to perceive information in complex international markets. These capabilities help overcome issues such as information asymmetry in international markets, providing a technological foundation for the internationalization of enterprises and improving their level of internationalization [30].

Based on the above analysis, the following hypothesis is proposed:

H2: The establishment of FTZs promote the international development of enterprises by enhancing their level of digital transformation.

#### 3.2.2. Resource allocation efficiency mechanism.

To achieve internationalization, enterprises must make optimal use of their limited resources. The establishment of FTZs can improve resource allocation efficiency by reducing the misallocation of capital and labor factors and by enhancing the efficiency of digital factor allocation [31]. Optimizing internal resource allocation is crucial for enterprises to maintain healthy development and enhance their international competitiveness [32]. By leveraging the convenience of The establishment of FTZs, enterprises can strictly control the quality, quantity, timing, and associated requirements of raw material supply. They can also align with international markets to establish efficient workflows and business standards, optimize production processes, implement refined cost management, and quickly match global supply and demand.

The establishment of FTZs optimizes the investment environment based on market economy principles, creates a favorable business environment, and builds a sound property rights protection system, which attracts foreign investors [4]. This, in turn, draws more foreign enterprises into the market. Foreign enterprises typically exhibit higher production efficiency, advanced technological capabilities, and mature management models. As the number of foreign enterprises increases, competition within the industry intensifies. A highly competitive market environment and the survival-of-the-fittest mechanism constrain opportunistic behaviors by business agents. FTZ policies also attract highly skilled talent with broader international perspectives and expertise, which improves management decision-making efficiency and makes investment decisions more prudent and rational. This helps enterprises avoid being eliminated from the market, promotes better resource allocation efficiency, and allows companies to better adapt to the competitive demands of the international market.

Based on this analysis, the following hypothesis is proposed:

H3: The establishment of FTZs promote the international development of enterprises by enhancing their resource allocation efficiency.

#### 3.2.3. Green innovation mechanism.

Due to the disadvantages faced by foreign entrants and their home countries, they are inevitably influenced by the host country’s institutional framework. The internationalization behavior of Chinese enterprises often raises legitimacy concerns among stakeholders in the host country [33]. To address this issue, Chinese enterprises can reshape the perceptions of host country stakeholders through their actions and gain external legitimacy [34]. In the current context, the rise of environmentalism has made green innovation strategies one of the most effective ways to enhance corporate social responsibility performance [35]. This demonstrates that enterprises are conforming to the host country’s institutional framework and signals an active effort to legitimize their operations. Both actions help alleviate legitimacy concerns among host country stakeholders, facilitating the smooth internationalization of the enterprise [36]. One of the significant obstacles to green innovation is the underdeveloped market process and the lack of innovative talent. However, the establishment of FTZs can address these challenges by improving market processes and fostering the concentration of innovative talent, thereby enhancing the level of green innovation within enterprises [37].

Based on this analysis, the following hypothesis is proposed:

H4: The establishment of FTZs promote the international development of enterprises by enhancing their green innovation capabilities

**Fig 1:**
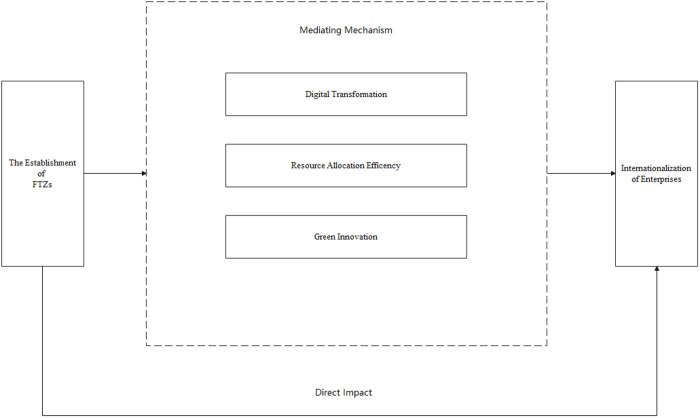
The influence mechanism of the establishment of FTZs on enterprise internationalization.

In summary, this paper presents a framework (Fig 1) that illustrates the impact mechanisms for how the establishment of FTZs affects international expansion of enterprises.

## 4. Empirical framework

### 4.1. Empirical specifications

In recent years, the Difference-in-Differences (DID) method has been widely used to evaluate the impact of policies [38]. Its fundamental principle is to treat policy implementation as a quasi-natural experiment outside the economic system and assess observed changes by constructing a counterfactual framework. The selection of FTZs is typically independent of individual firms, which helps mitigate the issue of reverse causality and enables a more precise identification of the impact of FTZs establishment on firms’ internationalization levels [39]. Therefore, applying the DID method to assess the policy effects of the establishment of FTZs in this study is appropriate. Accordingly, the following multi-period DID model is established (1):


$${\text{EI}}_{\text{i,t}}=\alpha_{0}+\alpha_{1}{\text{DID}}_{\text{i,t}}+{\alpha_{2}\text{Controlit}}_{\text{i,t}}+\lambda_{\text{i,t}}+\mu_{\text{i,t}}+\varepsilon_{\text{i,t}}$$ (1)

$${\text{DID}}_{\text{i,t}}={\text{treat}}_{\text{i,t}}*{\text{time}}_{\text{i,t}}$$

Let the firm be denoted as i and the year as t. The level of internationalization for firm i in year t is represented by EI_i,t_. The core explanatory variable is DID_i,t_, which represents the interaction term between the treatment group policy implementation dummy variable (treat_i,t_) and the time dummy variable (time_i,t_). The treatment group dummy variable (treat) is defined as follows: for companies registered within the FTZs, treat takes the value of 1; for companies registered outside the zone, treat takes the value of 0. The policy implementation time dummy variable (time) is set based on the different establishment times of FTZs in various batches. This study uses a multiple-time DID model, with different impact points defined according to the establishment dates of the respective FTZs. Taking the Shanghai FTZ, established in 2013, as an example, 2013 is the exogenous policy shock point. The time dummy variable takes 0 before 2013 and 1 after 2013. The same method is applied to other batches of FTZs.The total of all control variables is denoted by Control_it_; the fixed effect at the time level is denoted by λ_t_;the fixed effect at the industry level is denoted by μ_i_; the random disturbance term is represented by ε_it_. In the regression analysis, the study applies city-level clustering of standard errors to correct for heteroscedasticity, in order to eliminate issues arising from bias or regional differences.

This paper employs a mediation effect approach for testing to examine how the establishment of the FTZs influences corporate internationalization through its impact on digital transformation, resource allocation efficiency, and green innovation. Currently, the stepwise method remains the most popular testing approach. Therefore, based on model (1), this paper constructs mediation effect models (2) and (3).


$${\text{M}}_{\text{i,t}}=\alpha_{0}+\alpha_{1}{\text{DID}}_{\text{i,t}}+\alpha_{2}{\text{Controlit}}_{\text{i,t}}+\lambda_{\text{i,t}}+\mu_{\text{i,t}}+\varepsilon_{\text{i,t}}$$ (2)

$${\text{EI}}_{\text{i,t}}=\alpha_{0}+\alpha_{1}{\text{DID}}_{\text{i,t}}+\alpha_{2}{\text{M}}_{\text{i,t}}+\alpha_{3}{\text{Controlit}}_{\text{i,t}}+\lambda_{\text{i,t}}+\mu_{\text{i,t}}+\varepsilon_{\text{i,t}}$$ (3)

In these models, M represents the mediator variable, the specific definition of which will be provided later, and the control variables remain consistent with those in the previous model. Model (2) is used to verify the impact of the establishment of the FTZs (DID) on the mediator variable (M). At the same time, Model (3) is employed to test the mediation effect of M in the relationship between The establishment of FTZs (DID) and corporate internationalization (EI).

### 4.2. Data issues

This study focuses on A-share listed companies in China from 2009 to 2022 to explore the impact of the FTZs establishment on corporate internationalization. To ensure data reliability, firms categorized as ST or *ST, which had incomplete information disclosures or exhibited abnormal financial conditions, were excluded from the sample. A linear interpolation method addressed a small amount of missing data. The final dataset includes 2,244 A-share listed companies, resulting in 19,114 annual observations. The data sources include the CSMAR Database, CNRDS Database, and publicly disclosed annual reports from the listed companies.

### 4.3. Main variable definitions

#### 4.3.1. Independent variable DID_i,t_.

The explanatory variable in this study is DID_i,t_, which is a dummy variable representing the establishment of the FTZs. This variable is constructed using the address and name information of firms from the matched business database. If firm i is located in a city where the FTZs was established in year t, and the firm is situated within the FTZs area, the value is assigned as 1; otherwise, it is assigned as 0.

#### 4.3.2. Dependent variables EI.

Based on the research of previous scholars, corporate internationalization can be measured in terms of breadth and depth [40]. Some researchers have confirmed that internationalization breadth refers to the extent to which a company operates in more countries, indicating a broader scope of international expansion. This also implies that the company faces more complex and diverse market environments. Compared to other internationalization indicators, internationalization breadth better captures a company’s internationalization level and is considered a core dimension of international expansion [41]. Therefore, in this study, internationalization breadth is measured by taking the natural logarithm of the number of overseas subsidiaries a company has in a given year plus one. An alternative measure of internationalization breadth is used as a robustness check, which is the natural logarithm of the number of regions and countries covered by a company’s overseas subsidiaries in a given year plus one.

#### 4.3.3. Mediating variable M.

The mediating variables in this study are as follows:

(1) **Degree of Digital Transformation (M**_**1**_). In this study, following the approach of Zhai H. et al. [42], text analysis and word frequency statistics are conducted on the annual reports of listed companies. We use Python text mining ability to analyze and summarize the frequency of digital transformation related terms in the annual reports of sample companies, and then sum up the occurrence frequency of digital related keywords of each company to obtain the total digital word frequency, add 1 and take the natural logarithm, so as to construct the digital transformation index of the company.
(2) **Resource Allocation Efficiency (M**_**2**_). In this study, we adopt the measurement method proposed by Richardson S [43] and use the absolute value of the residuals from the model to assess resource allocation efficiency. A higher value indicates poorer resource allocation efficiency. Following Richardson S’s approach, we establish Equation (4) to first estimate the company’s reasonable level of investment for the year, and then calculate over-investment or under-investment to measure the company’s investment efficiency.

$$\begin{array}{c} {\text{Invest}}_{\text{nit}}=\beta_{0}+\beta_{1}{\text{Growth}}_{\text{nit}-1}+\beta_{2}{\text{Lev}}_{\text{nit}-1}+\beta_{3}{\text{Roa}}_{\text{nit}-1}+\beta_{4}{\text{Age}}_{\text{nit}-1}\\ +\beta_{5}{\text{Size}}_{\text{nit}-1}+\beta_{6}{\text{Invest}}_{\text{nit}-1}+\Sigma \text{Indu}+\Sigma \text{Year}+\varepsilon_{\text{nit}} \end{array}$$ (4)

In this model, fixed asset investment Invest_nit_ is the proportion of the original fixed asset value to the total assets at the beginning of the period; Growth_nit-1_ is the growth rate of the company’s main business revenue; the debt-to-asset ratio Lev_nit-1_ is the proportion of total liabilities to total assets; Roa_nit-1_ is the return on assets; Age_nit-1_ is the age of the company; Size_nit-1_ is the company’s total asset size, measured as the natural logarithm of total assets; and Invest_nit-1_ is the fixed asset investment of the company in year t − 1t-1t − 1, calculated in the same way as above. Indu and Year are dummy variables for industry and year, respectively.When the residual from Equation (4) is greater than 0, resource allocation efficiency M_2_ is equal to the residual, which measures the level of over-investment by the firm. If the residual is less than 0, indicating under-investment by the firm, M_2_ takes the absolute value of the residual, reflecting the degree of under-investment. Both over-investment and under-investment reflect the inefficiency of the company’s investment decisions. The lower the level of inefficient investment in a given year, the higher the company’s investment efficiency and resource allocation efficiency.

(3) **Level of Green Innovation (M**_**3**_). For the proxy variable of corporate green innovation level, this study follows the approach of Lin H et al. [44], using the number of green patent applications filed by the listed company in a given year as the indicator. In the empirical analysis, 1 is added to the count, and the natural logarithm is taken.

#### 4.3.4. Control variables.

Following the approach of previous literature [11,27,29], this study chose the following indicators as firm-level control variables (Table 1): (1) Firm Size (Size): Measured by the logarithm of the company’s total assets. (2) Return on Assets (ROA): Measured by the company’s return on net assets. (3) Capital Structure (Lev): Measured by the debt-to-asset ratio. (4) Ownership Concentration (Top 1): Measured by the proportion of shares held by the largest shareholder. (5) Listing Age (ListAge): Represented by the natural logarithm of the number of years since the company was listed. (6) Proportion of Independent Directors (Indr): Measured by the proportion of independent directors to the total number of directors. (7) Dual Role (One): A value of 1 if the chairman and CEO are the same person; otherwise, the value is 0. (8) Cash Flow (CashFlow): Measured by the ratio of net cash flow from operating activities to total assets. (9) Price-to-Book Ratio (PB): Measured by the company’s share price ratio to its book value per share.

**Table 1:** Description of main variables.

Variable Type	Variable Name	Variable Symbol	Variable Explanation
Dependent variables	Enterprise internationalization level	EI	Ln (number of overseas subsidiaries owned by the enterprise in the current year +1)
Independent variable	The establishment of FTZs	DID	If the FTZs system in the region where the enterprise is located has been implemented in the current year, the value is 1; Otherwise, the value is assigned to 0.
Mediating variable	Degree of Digital Transformation	M_1_	Ln (disclosure times of “digital” keywords +1)
	Resource Allocation Efficiency	M_2_	The lower the level of inefficient investment of the enterprise in that year, the higher the investment efficiency and resource allocation efficiency.
	Level of Green Innovation	M_3_	Ln(number of green patent applications of listed companies in the current year +1).
Control variable	Firm Size	Size	Ln(total assets +1)
	Return on Assets	ROA	Measured by the company’s return on net assets.
	Capital Structure	Lev	Measured by the debt-to-asset ratio.
	Ownership Concentration	Top1	Measured by the proportion of shares held by the largest shareholder.
	Listing Age	ListAge	Ln(Year of the current year – year of listing +1)
	Proportion of Independent Directors	Indr	Measured by the proportion of independent directors to the total number of directors.
	Dual Role	One	A value of 1 if the chairman and CEO are the same person; otherwise, the value is 0.
	Cash Flow	CashFlow	Measured by the net cash flow ratio from operating activities to total assets.
	Price-to-Book Ratio	PB	Measured by the company’s share price ratio to its book value per share.

### 4.4. Descriptive statistics

The descriptive statistics of the research variables in this study are presented in Table 2. Among them, the mean value of EI is 0.511, with a maximum value of 4.615 and a minimum value of 0. This indicates that the overall level of internationalization among domestic companies is relatively low, with significant variation. Additionally, as shown in Table 2, the fluctuations of the control variables are generally within a reasonable range.

**Table 2:** Descriptive statistical results.

Variable	Observation	Mean	S D	Min	Max
EI	19,115	0.511	0.799	0	4.615
DID	19,115	0.373	0.484	0	1
M_1_	19,115	1.456	1.393	0	6.151
M_2_	15,263	0.129	0.249	0	14.470
M_3_	18,755	0.546	0.983	0	7.523
Size	19,115	22.450	1.666	18.760	31.310
ROA	19,115	0.112	0. 079	−0.898	1.426
Lev	19,115	0.398	0.206	0.008	0.966
Top1	19,115	0.363	0.154	0.036	0.900
ListAge	19,115	1.831	0.974	0	3.497
Indr	19,115	0.375	0.055	0.1	0.8
One	19,115	0.298	0.457	0	1
CashFlow	19,115	0.062	0.073	−0.744	0.726
PB	19,115	3.553	2.949	−1.453	53.637

## 5. Estimation results

### 5.1. Baseline results

The present study employs a regression model (1) to test Hypothesis H1 using the least squares method, analyzing the impact of the establishment of FTZs on the internationalization level of enterprises. The baseline regression results are presented in Table 3. Robust standard errors clustered by the city are utilized to address potential issues related to serial correlation and heteroscedasticity while controlling for time effects, industry-fixed effects, and other control variables stepwise in the regression analysis. Specifically, columns (1) and (3) display the regression outcomes without control variables and a series of control variables included, respectively, along with controls for time-fixed and industry-fixed effects. The coefficient representing the impact of FTZs establishment on enterprise internationalization levels is 0.091, which is statistically significant at the 1% level. This indicates that after establishing free trade experimental zones, enterprises experience an average annual increase in their internationalization levels by approximately 9.1%.

**Table 3:** Results of the benchmark regression analysis.

	(1) EI	(2) EI	(3) EI
DID	0.245 *** (7.16)	0.171 *** (5.34)	0.091 *** (2.76)
Size		0.176 *** (6.88)	0.288 *** (13.71)
ROA		0.114 (0.75)	−0.002 (−0.01)
Lev		−0.242 *** (−3.09)	0.059 (0.67)
Top1		−0.303 *** (−2.67)	−0.277*** (−3.11)
ListAge		0.025** (2.05)	−0.013 (−1.00)
Indr		0.379 * (1.82)	0.053 (0.25)
One		0.156 *** (5.85)	0.088 *** (3.27)
CashFlow		0.405 *** (3.13)	0.385 *** (3.08)
PB		0.011 *** (2.62)	0.10 ** (2.29)
Constant	0.419 *** (20.77)	−3.613*** (−6.13)	−6.002 *** (−11.92)
Industry FE	NO	NO	YES
Year FE	NO	NO	YES
R^2^	0.022	0.130	0.266
Adj-R^2^	0.022	0.129	0.262
Observation	19,115	19,115	19,114

Note: The data in brackets are standard errors, and the robust standard errors are clustered to the city dimension; ***, **, and * indicate the significance levels of 1%, 5%, and 10%, respectively, and the same below

This empirical result holds substantial practical and economic significance as it confirms the critical role of the establishment of FTZs in enhancing enterprise internationalization levels, effectively promoting Chinese listed companies’ development of internationalization of initiatives. Despite the currently low overall internationalization levels among China’s listed companies—significantly lagging internationally leading firms—the positive effects of FTZs construction have become particularly crucial. By implementing a strategy centered around FTZs, enterprises have significantly accelerated their development of internationalization of processes. In summary, research findings validate that the establishment of FTZs can significantly enhance enterprise internationalization levels, thus confirming Hypothesis H1.

### 5.2. Parallel trends assumption test

This study identifies the effect of the establishment of FTZs on corporate internationalization using the multiple-period difference-in-differences (DID) method. The prerequisite for applying the multiple-period DID method is that the treatment and control groups exhibit parallel trends before the policy shock, meaning that the change in the internationalization level of firms in pilot regions is parallel to that of firms in non-pilot regions. Based on this, the following model (5) is constructed:


$${\text{EI}}_{\text{i,t}}=\alpha_{0}+\Sigma_{7}^{4}\alpha_{n}{\text{DID}}_{\text{i,t}}+\alpha_{3}{\text{Controlit}}_{\text{i,t}}+\lambda_{\text{i,t}}+\mu_{\text{i,t}}+\varepsilon_{\text{i,t}}$$ (5)

**Fig 2:**
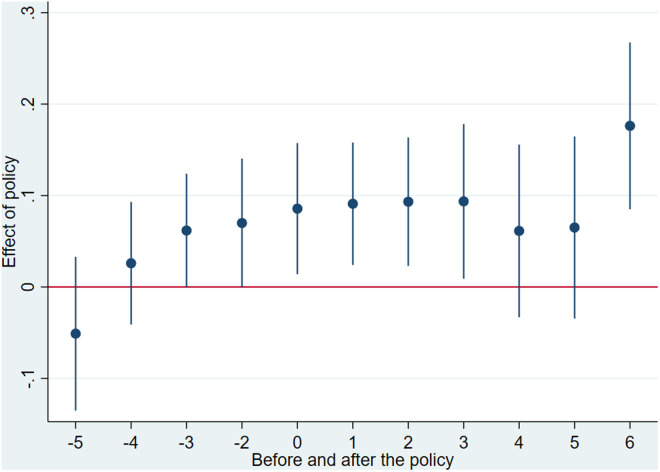
Parallel trend test chart.

In this model, DID_i,t_ represents a set of dummy variables. The value is 1 if the FTZs was established in the region where firm iii is located in year t, and 0 before the policy implementation. The other variables have the same meanings as in Equation (1), and the regression results are shown in Fig 2. $\alpha_{n}$represents a series of estimated values for the years before and after the establishment of FTZs. Since the first FTZ in China was established in 2013, there have been fewer observations at both ends. Therefore, following the approach of Li et al. [45], this study combines observations that occurred more than 5 years before the establishment of FTZs into the −5 period and those occurring more than 6 years after the establishment into the 6 period. Thus, the range of k is [−5,6]. Additionally, the estimation process excludes observations for the −1 period, which represents the year immediately before the establishment of FTZs, to address the dummy variable trap issue [5].

As shown in Fig 2, the coefficients before the establishment of FTZs are not statistically significant, indicating that there was no significant difference in the internationalization levels between the treatment and control groups before the policy implementation. After the establishment of the FTZs, the coefficients became significantly positive, suggesting that the policy significantly enhanced the internationalization level of firms, and its effects persisted for a considerable period. This satisfies the assumption of parallel trends.

### 5.3. Placebo test

**Fig 3:**
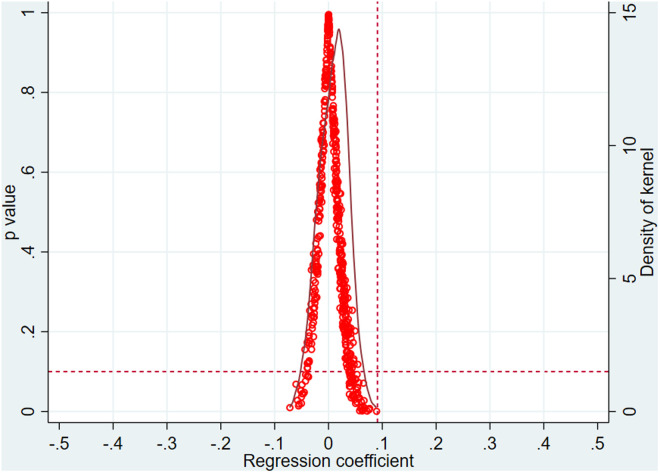
Placebo test chart.

To further verify whether the estimated results of the core explanatory variable in the baseline regression adequately control for the influence of factors other than the FTZs on corporate internationalization [46], this study employs a placebo test to assess the likelihood of a random effect of the establishment of FTZs. A placebo test is conducted by repeating the process 500 times, with each iteration randomly selecting a group of firms to serve as a placebo treatment group. The detailed results are shown in Fig 3. As seen in Fig 3, the regression coefficients estimated from the random samples are normally distributed around zero, and all coefficient values are lower than the baseline coefficient of 0.091. The placebo test, generated through random sampling, eliminates the potential interference of firm-specific factors, thereby confirming the reliability of the baseline regression results.

### 5.4. Robustness test and endogeneity treatment

#### 5.4.1. PSM-DID.

Although the baseline regression controls for time and industry-fixed effects, there may still be some selection bias between the treatment and control groups, potentially leading to estimation errors in the main conclusions. This study employs the propensity score matching (PSM) method for robustness testing to address endogeneity issues arising from omitted variables and sample selection bias.

Specifically, we use a Logit model to calculate propensity scores and mitigate bias. The samples are matched using three approaches: nearest neighbor matching (n = 1, without replacement), caliper matching (n = 1, caliper value = 0.02), and kernel matching. After matching, we retain the matched samples and the treatment group and re-run the regression analysis. As shown in Table 4, the results of the PSM-DID test remain statistically significant, confirming the robustness of the baseline regression findings.

**Table 4:** PSM-DID.

Variable	(1) Nearest neighbor matching	(2) Nearest neighbor matching with caliper	(3) Nuclear Matching
DID	0.056 * (1.67)	0.074 ** (2.23)	0.083 ** (2.18)
Control variables	YES	YES	YES
Constant	−5.275 *** (−11.24)	−5.625 *** (−14.27)	−6.488 *** (−10.34)
Industry FE	YES	YES	YES
Year FE	YES	YES	YES
R^2^	0.239	0.255	0.275
Adj-R^2^	0.233	0.249	0.270
Observations	13,002	12,955	12612

#### 5.4.2. Changing the measurement method of the explanatory variable.

Since companies may establish multiple subsidiaries in a single country or region to gain advantages for their development, it is important to avoid misjudging their internationalization level due to such behavior. To address this, this study follows existing research and uses the number of countries where a company’s overseas subsidiaries are located to measure its internationalization level. This variable is then used as the dependent variable to re-estimate the baseline model. The results, shown in Column (1) of Table 5, indicate that the DID coefficient is 0.075 and significant at the 1% level, confirming the robustness of the baseline regression results.

**Table 5:** Robustness test of changing the measurement method of explanatory variables.

Variable	(1) Replacement of Dependent variables	(2) High dimensional fixed effects and interaction fixed effects	(3) Eliminate some samples
DID	0.074 *** (2.71)	0.036 * (1.68)	0.085 ** (2.05)
Control variables	YES	YES	YES
Constant	−4.827 *** (−13.40)	−4.683 *** (−6.97)	−6.242 *** (−12.05)
Industry FE	YES	NO	YES
Year FE	YES	NO	YES
Time-Industry FE	NO	YES	NO
Individual fixed effects	NO	YES	NO
R^2^	0.262	0.810	0.289
Adj-R^2^	0.258	0.773	0.284
Observations	19,114	18,988	15,125

#### 5.4.3. Inclusion of industry-time interaction fixed effects.

When conducting regression analysis using the Difference-in-Differences (DID) method, controlling for individual fixed effects that vary over time helps reduce omitted variable bias, making the regression results more robust [47]. Therefore, this study introduces an interaction term between time and industry, along with firm fixed effects, to mitigate the fluctuations in results caused by endogeneity issues and to conduct robustness checks. The regression results in Column (2) of Table 5 show that after controlling for “time-industry interaction fixed effects” and “firm-specific fixed effects,” the coefficient of the FTZs policy is significantly positive at the 10% confidence level. This suggests that the establishment of FTZs can enhance the level of corporate internationalization, and the conclusion is robust.

#### 5.4.4. Exclusion of certain samples.

The variation in sample periods may also affect the regression results. Considering that the municipalities directly under the central government in the sample have higher levels of investment freedom, trade openness, regulatory transparency, fairness and efficiency of supervision, and a more favorable business environment, these factors may influence the baseline regression results. Additionally, as China’s only provincial-level FTZ, the Hainan Free Trade Port is highly unique, with significant differences from other FTZs in terms of geographical location, policy support, and industrial positioning. Therefore, this study excludes enterprises in the municipalities directly under the central government and the Hainan Free Trade Port for robustness checks. The regression results in Column (3) of Table 5 still show a significant coefficient, indicating that the conclusions remain unchanged and the results are robust.

#### 5.4.5. instrumental variable.

Although this study controls for firm fixed effects, there may still be endogeneity issues due to omitted variables. To address this, each region’s average nighttime light radiation pixel intensity for the given year is used as an instrumental variable for whether a city is included in the FTZs pilot program.

Regions such as Shenzhen, Zhuhai, Shantou, and Xiamen, followed by the establishment of the Shanghai FTZ in 2013, have long been leaders in opening up to the outside world, with areas like Shanghai and Guangdong showing high nighttime light intensity. This makes them a suitable instrument in terms of relevance. Second, nighttime light intensity in cities does not directly influence a firm’s internationalization decisions, meeting the exogeneity requirement for an instrument.

In the estimation, an interaction term between city nighttime light intensity and time dummy variables (NI) is constructed as the instrument for the establishment of FTZs. The first-stage regression results show that the instrument’s coefficient is significantly positive at the 1% level. In Table 6, the F-statistic is 15966.32 which exceeds the threshold of 16.38, indicating that the chosen instrument is not weak. Thus, we reject the null hypothesis that the instrument is weak, suggesting that the instrument selection is reasonable.

**Table 6:** instrumental variable.

Variable	(1) DID	(2) EI
NI	0.024 *** (126.36)	
DID		0.177 *** (9.26)
Control variables	YES	YES
Industry FE	YES	YES
Year FE	YES	YES
Kleibergen Paap·rk·		4083.76 ***
Kleibergen-Paap Wald rk F		15966.32 [16.38]
Observations	19114	19114

The second-stage regression results show that, even after accounting for endogeneity, the establishment of FTZs still significantly promotes firms’ foreign direct investment.

### 5.5. Mediation mechanism testing

The theoretical analysis in the previous sections suggests that the establishment of FTZs can enhance corporate digital transformation, which in turn promotes firm internationalization. To test hypotheses H2, H3, and H4, this study uses a stepwise approach based on models (2) and (3). Specifically, we examine whether the establishment of FTZs enhances corporate internationalization by promoting digital transformation, improving resource allocation efficiency, and fostering green innovation. Table 7 presents the results of the mediation mechanism tests.

**Table 7:** Mediation effect of green innovation.

Variable	(1) M_1_	(2) EI	(3) M_2_	(4) EI	(5) M_3_	(6) EI
DID	0.186*** (3.76)	0.035*** (3.12)	−0.014*** (−2.69)	0.108*** (2.94)	0.062** (2.02)	0.091*** (2.68)
M		0.085** (2.53)		−0.055** (−2.40)		0.033** (2.37)
Control variables	YES	YES	YES	YES	YES	YES
Constant	−2.416*** (−6.22)	−5.918*** (−11.59)	0.0002 (0.00)	−6.184*** (−12.00)	−8.240*** (−9.51)	−5.815*** (−10.56)
Industry FE	YES	YES	YES	YES	YES	YES
Year FE	YES	YES	YES	YES	YES	YES
R^2^	0.519	0.268	0.046	0.271	0.373	0.268
Adj-R^2^	0.516	0.264	0.040	0.266	0.370	0.264
Observations	19,114	19,114	15,262	15,262	18,754	18,754

#### 5.5.1. Mediation effect of digital transformation.

This study uses Equation (2) to analyze the impact of the establishment of FTZs on corporate digital transformation. According to Column (1) of Table 7, the estimated coefficient for the effect of the establishment of FTZs on the digital transformation (M_1_) level is 0.186, which is significantly positive at the 1% level. This confirms that the establishment of FTZs promotes corporate digital transformation. After incorporating the mediator variable, digital transformation level, we use Equation (3) to test the relationship between digital transformation and corporate internationalization. Column (2) of Table 7 shows that the impact of digital transformation on corporate internationalization is significantly positive at the 5% level, with an estimated coefficient of 0.085. This indicates that digital transformation plays a partial mediating role in the relationship between the establishment of FTZs and corporate internationalization. Therefore, hypothesis H2 is supported.

#### 5.5.2. Mediation effect of resource allocation efficiency.

This study uses Equation (2) to analyze the effect of the establishment of FTZs on corporate resource allocation efficiency. According to Column (3) of Table 7, the estimated coefficient for the effect of the establishment of FTZst on resource allocation efficiency (M_2_) is −0.014, which is significantly negative at the 1% level. This suggests that the establishment of FTZssignificantly reduces inefficiency in corporate investment and improves resource allocation efficiency. After incorporating resource allocation efficiency as a mediator, Column (4) of Table 7 shows that the coefficient for inefficiency in investment is significantly negative and significantly correlated at the 5% level. This implies that lower levels of inefficient investment reduce the internationalization level of firms, the higher the resource allocation efficiency, the higher the internationalization level of the firm. This indicates that the establishment of FTZs enhances corporate internationalization through the path of improving resource allocation efficiency. Thus, hypothesis H3 is supported.

#### 5.5.3. Mediation effect of green innovation.

This study uses Equation (2) to analyze the impact of the establishment of FTZs on corporate green innovation levels. According to Column (5) of Table 7, the estimated coefficient for the effect of the establishment of FTZs on green innovation (M_3_) is 0.062, which is significantly positive at the 5% level. This suggests that the establishment of FTZs significantly enhances corporate green innovation. After incorporating green innovation as a mediator, Column (6) of Table 7 shows that green innovation is significantly positively related to corporate internationalization at the 5% level. This confirms that the establishment of FTZs improves corporate internationalization through the channel of enhancing green innovation. Therefore, hypothesis H4 is supported.

### 5.6. Heterogeneity analysis

Due to the diversity of enterprise types, this paper examines the heterogeneity in the impact of the establishment of FTZs on enterprises’ internationalization levels from urban hierarchy, enterprise scale, factor intensity, corporate governance and technological attributes. This analysis aims to deepen our understanding of this issue.

#### 5.6.1. City-level heterogeneity analysis.

Regarding urban hierarchy. As a socialist market economy system, the establishment of FTZs in China may affect enterprises’ internationalization levels depending on their urban hierarchies. Although enterprises in higher-tier cities benefit from greater resource allocation efficiency, they also face more severe resource misallocation issues. In this context, ordinary city enterprises may gain more capital and labor resources through the institutional dividends provided by FTZs, leading to a more pronounced improvement in their internationalization levels. In this study, we categorize urban tiers as ordinal variables: ordinary sub-provincial cities are assigned a value of 1; municipalities directly under central authority are assigned 2; non-sub-provincial provincial capitals are assigned 3; and prefecture-level cities are assigned 4. Subsequently, we define samples with an urban tier greater than or equal to 3 as low-tier sample groups and those less than or equal to 2 as high-tier sample groups for grouped regression analysis. The results in Table 8, columns (1)-(3) indicate that the regression coefficients for low-tier city sample groups are significantly positive. In comparison, those for high-tier city sample groups are smaller and insignificant. These findings suggest that implementing the establishment of FTZs has a more evident promoting effect on the internationalization level of ordinary city enterprises.

**Table 8:** Heterogeneity analysis 1.

Variable	(1) Lower-Level Cities	(2) Higher-Level Cities	(3) Small Enterprises	(4) Large Enterprises	(5) Labor-Intensive	(6) Capital-Intensive	(7) Technology-Intensive
DID	0.110* (1.95)	0.065 (1.63)	0.052 (1.52)	0.138*** (2.87)	0.033 (0.80)	0.175** (2.43)	0.092* (1.94)
Control variables	YES	YES	YES	YES	YES	YES	YES
Constant	−5.830*** (−11.30)	−6.411*** (−8.92)	−3.226*** (−6.36)	−6.963*** (−9.12)	−4.625*** (−8.07)	−7.140*** (−10.12)	−6.906*** (−10.71)
Industry FE	YES	YES	YES	YES	YES	YES	YES
Year FE	YES	YES	YES	YES	YES	YES	YES
R^2^	0.312	0.275	0.121	0.275	0.251	0.342	0.263
Adj-R^2^	0.305	0.268	0.112	0.268	0.243	0.334	0.259
Observations	9,387	9,724	9,557	9,555	5,985	3,868	9,261

#### 5.6.2. Heterogeneity analysis of enterprise size.

Concerning enterprise scale. In this paper, according to the total assets, enterprises larger than the median are classified as large enterprises, and those smaller than the median are classified as small enterprises for estimation. As shown in Table 8, columns (3)-(4), DID coefficients are only significantly positive among large-sized enterprises. This phenomenon can be attributed to several factors: small-scale enterprises typically lack economies of scale and cannot secure sufficient external funding necessary for supporting their international expansion; they often possess relatively lower risk resistance capabilities, which makes them vulnerable to bankruptcy crises—thus diminishing their willingness towards international expansion; maintaining stability within domestic markets poses enough challenges for many small firms without adding further risks associated with globalization efforts. Conversely, large-sized firms possess adequate resources and capabilities to tackle challenges posed by global markets while benefiting from diversification strategies that mitigate market risks by entering new markets where they can acquire additional customers and revenue opportunities. Therefore, it is evident that the establishment of FTZs exerts more substantial promotional effects on large-sized firms’ internationalization.

#### 5.6.3. Heterogeneity analysis of factor intensity.

Regarding factor intensity considerations, the influence exerted by FTZ establishments varies across different intensities related to production factors affecting companies’ levels of internationalization as well. This study references industry classification standards set forth by regulatory authorities alongside relevant research [48], categorizing industries into technology-intensive, capital-intensive, and labor-intensive sectors before conducting group tests. The results displayed within Table 8 columns (5)-(7)indicate that the establishment of FTZs yields significant positive impacts upon the technological and capital-intensive sectors firm’s degree of internationalization; however, no notable effect was observed amongst labor-intensive sectors. This discrepancy likely arises because pursuing an internationally oriented development strategy necessitates specific knowledge and financial thresholds aligning closely with characteristics inherent within high-capita-focused businesses and high-tech-focused businesses. Labor costs might lack competitive advantages when competing globally since numerous labor-intensive organizations rely heavily upon inexpensive workforces whose benefits could diminish elsewhere around the globe. Additionally, labor-intensive sectors generally exhibit lower profit margins, making them increasingly cautious about incurring substantial expenses tied to expansion into foreign markets.

#### 5.6.4. Heterogeneity analysis of corporate governance.

From the corporate governance perspective, the upper echelons theory suggests that individual characteristics of executives—such as age, gender, experience, and personality—shape their values and cognitive abilities, which in turn influence their strategic choices [49]. Significant differences exist between the economic, institutional, and cultural environments of overseas regions and China, and the East and West management philosophies are also notably distinct. Overseas work experience and international education are crucial in shaping an executive’s management style, consequently affecting the company’s business decisions.

This study assigns a value of 0 to executives with overseas backgrounds and 1 to those without and conducts a heterogeneity test based on executives’ overseas backgrounds. Columns (1) and (2) of Table 9 show that the establishment of FTZs significantly positively impacts the internationalization levels of firms with executives from overseas backgrounds. However, this effect is noticeably weaker for firms without overseas executives. This may be because executives with overseas backgrounds typically have experience working in multinational companies or studying abroad, enabling them better to understand global markets’ competitive landscape and business models. Additionally, they tend to possess higher risk tolerance and a greater willingness to explore new market opportunities.

**Table 9:** Heterogeneity analysis 2.

Variable	(1) Executives with overseas background	(2) No senior executive with overseas background	(3) High-tech enterprises	(4) Non-high-tech enterprises
DID	0.141*** (3.31)	0.057* (1.85)	0.108*** (2.85)	0.073 (1.52)
Control variables	YES	YES	YES	YES
Constant	−6.870*** (−9.27)	−5.000*** (−10.52)	−6.785*** (−14.51)	−5.046*** (−8.15)
Industry FE	YES	YES	YES	YES
Year FE	YES	YES	YES	YES
R^2^	0.327	0.237	0.272	0.262
Adj-R^2^	0.315	0.231	0.269	0.254
Observations	5,026	14,083	11,082	8,024

#### 5.6.5. Heterogeneity analysis of science and technology attributes.

From the perspective of technological attributes, the sample companies are classified into high-tech and non-high-tech enterprises according to the “High-Tech Industry Classification (2017)” for the heterogeneity test. Columns (3) and (4) of Table 9 show significant heterogeneity in the internationalization of high-tech and non-high-tech companies, with the positive impact being notably higher for high-tech companies compared to non-high-tech companies.

High-tech enterprises typically require large-scale investments in production and R&D. Internationalization helps these companies expand their markets and increase output, thereby achieving economies of scale and distributing the costs of R&D and production. Furthermore, innovation in the high-tech industry relies heavily on global scientific research collaboration and knowledge flow. Through internationalization, high-tech companies can collaborate with leading research institutions, universities, and other firms worldwide, sharing technological innovations and R&D resources.

## 6. Analysis on the spatial spillover effect of enterprise internationalization

From a geographical perspective, FTZs exhibit significant spatial externalities and geopolitical advantages. Consequently, implementing related policies may generate spillover effects on surrounding regions. Research conducted by scholars [50] on FTZ construction plans has revealed that enterprises within FTZs experience export spillover effects, which enhance the export performance of non-FTZ enterprises. Notably, these spillover effects are more pronounced among larger-scale firms, those with higher productivity, or state-owned non-FTZ exporters. Such spillover effects may influence the internationalization level of the enterprises studied in this paper.

Companies operating within FTZs often engage in exchanges and collaborations with neighboring businesses. This interaction allows nearby companies to learn from those in FTZs through various forms of partnership. Such dynamics facilitate the sharing of internationalization experiences and strategies from FTZs enterprises to their counterparts in adjacent areas.

The capital and investments generated by the establishment of FTZs not limited to movements within the zone itself. When investors identify high-quality firms in nearby regions, they may also provide financial support beyond the boundaries of the FTZs. The establishment of FTZs not only attracts advanced technology but also draws managerial talent, especially individuals with international experience. This can lead to increased talent mobility, benefiting surrounding areas. Consequently, this influx of skilled professionals is likely to enhance the human capital of local businesses, further promoting their internationalization efforts.

This study aims to systematically examine the spatial spillover effects associated with the establishment of FTZs. To determine whether these zones influence the levels of enterprise internationalization due to the establishment of FTZs, we excluded provinces that do not have any established FTZs. We then conducted regression analysis on cities that have implemented FTZs within the remaining sample. A decrease in the significance level of the regression coefficients compared to the primary regressions would suggest that the technological advancements resulting from policy implementation exhibit spatial spillover characteristics.

As indicated in Column (1) of Table 10, the difference-in-differences (DID) coefficient decreased from 0.91 to 0.83, while still remaining positive at a 5% significance level. Although this represents a reduction compared to the primary regression’s significance level of 1%, it preliminarily supports the idea that the establishment of FTZss do exert spatial spillover effects on enterprise internationalization levels.

**Table 10:** Spatial spillover effect of enterprise internationalization.

Variable	(1) Excluding provinces without free trade zones	(2) Cities with free trade zones are excluded
DID	0.083** (2.47)	0.113*** (2.65)
Control variables	YES	YES
Constant	−6.220*** (−12.03)	−5.647*** (−8.94)
Industry FE	YES	YES
Year FE	YES	YES
R^2^	0.276	0.312
Adj-R^2^	0.272	0.303
Observations	17,771	6,500

Furthermore, we removed cities designated as trial zones for additional analysis, focusing on regression at the provincial level. If provinces hosting an FTZ continue to significantly impact the dependent variables after excluding trial zone cities, this would further confirm the existence of spatial spillover effects. The results presented in Column (2) of Table 10 demonstrate that, with a 1% significance level, the DID coefficients remain significant, further validating that FTZs policy promote enterprise internationalization through observable spatial spillovers.

## 7. Estimation results

As a significant institutional innovation, the FTZ is tasked with serving as a new gateway for China’s foreign trade and investment. Our study provides new empirical evidence on the impact of FTZs on firms’ development of internationalization of strategies, contributing to a more comprehensive and in-depth understanding of the specific effects that the establishment of FTZs has on micro-level enterprises.

This study takes a macro-micro intersection perspective to examine the policy effects resulting from the establishment of FTZs within the new development framework. Utilizing data from Chinese A-share listed companies between 2009 and 2022, we empirically analyze the impact of the establishment of FTZs on firms’ levels of internationalization and the mechanisms at play.

The findings indicate that FTZs significantly enhance firms’ international expansion. This conclusion holds even after addressing and resolving various endogeneity issues. The establishment of FTZs exerts a notable positive effect on firms’ internationalization levels, contributing to greater external openness. This effect remains robust after conducting several endogeneity and robustness tests.

Furthermore, digital transformation, resource allocation efficiency, and green innovation mediate this process. Our analysis reveals that FTZs facilitate firms’ overseas expansion by promoting digital transformation, improving resource allocation efficiency, and encouraging green innovation.

Additionally, a heterogeneity analysis shows that the impact of the establishment of FTZs on the enhancement of internationalization levels is especially pronounced among enterprises in low city levels, large-sized companies, capital-intensive and technology-intensive companies, businesses with executives who have overseas experience, and high-tech companies. Further research indicates that the positive effects of the establishment of FTZs on firm internationalization also extend to surrounding cities and provinces, demonstrating spatial spillover effects.

These findings provide compelling micro-level evidence for China’s strategy of promoting outward expansion through FTZs. They offer valuable insights for other emerging economies that are gradually implementing internationalization strategies for their firms.

Based on the above research findings, this study offers the following recommendations to better leverage the establishment of FTZs in promoting the internationalization of firms:

**Government should formulate scientific policies to promote regional enterprise internationalization:**

1. The government should recognize the crucial role of FTZs in enhancing corporate internationalization. It is essential to coordinate further the national strategy for opening up and related policies, fostering a more open business environment to facilitate firms’ global expansion. Accelerating alignment with high-standard international trade and investment rules and promoting institutional innovations—such as trade facilitation, investment liberalization, and financial openness—will be key. Additionally, improving government efficiency, streamlining administrative procedures, and reducing institutional barriers to international business operations will further support firms in their globalization efforts.
2. The government should acknowledge the mediating role of digital transformation, resource allocation efficiency, and green innovation in advancing corporate internationalization. Targeted policies should be developed to promote digitalization, enhance resource efficiency, and encourage corporate innovation. Moreover, local governments should be urged to actively implement these policies to create a robust digital ecosystem conducive to firms’ international expansion. To cater to different types of enterprises (e.g., high-tech firms, SMEs), differentiated support policies—such as tax incentives, financing assistance, and international market expansion services—should be introduced to facilitate their globalization strategies.
3. The government must also recognize the spatial spillover effects of the establishment of FTZs on surrounding cities and provinces. Strengthening regional collaboration is essential to foster collective corporate internationalization. Efforts should be made to enhance coordination between FTZs and neighboring regions, encouraging enterprises in non-FTZ cities to learn from FTZs experiences and improve their global competitiveness. Establishing regional international cooperation platforms will be beneficial in promoting overseas market expansion for enterprises in coastal, inland, and border areas, further integrating them into the global economy.

**Enterprises should enhance their awareness of internationalization to support their own development:**

1. Businesses should create effective strategies to enhance their globalization efforts while also increasing awareness of social responsibility and improving service capabilities. Given the rapidly changing conditions of the global economy, companies must adopt a proactive approach by developing market-responsive strategies tailored to the specific characteristics and cultural differences of their target markets. Utilizing the policy advantages provided by FTZs (FTZs) will help strengthen product competitiveness in those markets and capitalize on opportunities arising from globalization initiatives.
2. Enterprises should actively promote resource sharing, technology collaboration, and benefit maximization through digital technology. They should focus on creating innovative products and services that meet the needs of their target market in order to achieve differentiated competitive advantages. At the same time, it is essential to optimize resource allocation and improve production process efficiency. This includes implementing refined cost management, achieving rapid alignment of global supply and demand, and further enhancing production efficiency to strengthen market competitiveness. Additionally, businesses must recognize the importance of social responsibility, particularly in the research and development of green innovation technology. This involves focusing on energy conservation, emission reduction, waste management, and efficient resource utilization while fulfilling their social responsibilities. By doing so, companies can improve corporate governance, effectively respond to external challenges, mitigate competitive disadvantages in both source and target countries, and enhance their popularity and influence in the international market.
3. Businesses outside the FTZs should acknowledge the spatial spillover effects of the FTZs policy on nearby cities and provinces. They should actively leverage the capital and investments that flow out of the zone, while also learning from and emulating the internationalization strategies and experiences of enterprises within the zone. Additionally, it’s important to attract high-end technical and management talent to build human capital, promote the enhancement of human capital structures, and strengthen international development efforts. Moreover, these enterprises should actively support the development of internationalization of strategy, pursue market diversification to reduce reliance on a single market, mitigate risks, and better adapt to various market changes and challenges.

Although this study expands the boundaries of research on the microeconomic impacts of FTZs, it still has some limitations. Due to the lack of more detailed data, we assumed that all firms within FTZs benefit from policy incentives, whereas, in reality, these incentives may vary across specific regions within the pilot cities. Additionally, we only explored the impact of FTZs on the international breadth of firms without investigating their effect on the depth of internationalization. Therefore, we aim to gradually refine and improve our work in this area in future research.

## Supporting information

S1 Data The batches of the establishment of FTZs. (DOCX)

S2 Data The digital transformation of enterprises. (XLSX)
