# Supplementary material for: Can the establishment of free trade zones promote the internationalization of enterprises? – Evidence from micro-enterprise level
Source: PLoS One. 2025 Jul 16;20(7):e0322125. doi: 10.1371/journal.pone.0322125 (PMC12266465; doi:10.1371/journal.pone.0322125)
Supplement: S1 Data — (DOCX) [file pone.0322125.s001.docx]

**FTZ**

**2013：**Shanghai city

2015：Tianjin city；

Guangdong Province： Shenzhen city； Guangzhou city； Zhuhai city

Fujian Province：Xiamen city, Fuzhou city

2017：Chongqing city

Sichuan Province：Chengdu city； Luzhou city

Hubei Province：Yichang city, Wuhan city; Xiangyang city

Shannxi Province：Xian city, Xianyang city,

Liaoning Province：Dalian city, Shenyang city, Yingkou city,

Zhejiang Province：Zhoushan city,

Henan Province：Luoyang city, Kaifeng city

2018: Hainan Province

2019: Hebei Province：Shijiazhuang city, Tangshan city, Langfang city, Baoding city,

Shandong Province：Jinan city, Qingdao city, Yantai city,

Yunnan Province：Kunming city,

Jiangsu Province：Suzhou city, Nanjing city, Lianyungang city,

Guangxi Province：Nanning city,

Heilongjiang Province：Haerbin city

2020: Beijing city

Anhui Province：Hefei city, Wuhu city, Bengbu city,

Zhejiang Province：Hangzhou city, Ningbo city, Jinhua city, Yiwu city,

Hunan Province：Changsha city, Yueyang city, Chenzhou city

2023: Xinjiang Province: Urumchi city, Kashgar city, Khorgos city
